# Spatio-Temporal Distribution Characteristics of Syphilis: on the Scale of Towns (Streets) in Nantong City, Jiangsu Province, China

**DOI:** 10.3389/ijph.2025.1606875

**Published:** 2025-03-18

**Authors:** Zhihai Zhang, Xiaoyan Hou, Maomao Liu, Maoxuan Wu, Ping Zhu, Xiaoyi Zhou

**Affiliations:** Department of STD and AIDS Control and Prevention, Nantong Center for Disease Control and Prevention, Nantong, Jiangsu, China

**Keywords:** syphilis, spatial autocorrelation, spatio-temporal scanning, cluster, epidemiology

## Abstract

**Objectives:**

To investigate the spatio-temporal distribution characteristics and changing trends of syphilis in Nantong city.

**Methods:**

Joinpoint regression model, spatial autocorrelation and SaTScan spatio-temporal scanning were used to analyze the trend of syphilis reported incidence and spatio-temporal distribution characteristics in Nantong City.

**Results:**

From 2013 to 2022, the reported incidence of syphilis in Nantong City increased at an average annual rate of 6.60%, of which the increase rate of latent syphilis was 13.45%. The high-high clustering areas were mainly distributed in 15 streets of Chongchuan District and all streets of Nantong Development Zone. SaTScan spatio-temporal scanning detected a total of two clustering areas, all from 2021 to 2022. The first cluster includes 24 streets with a radius of 16.27 km, and the second cluster includes 18 streets within a radius of 34.90 km.

**Conclusion:**

The reported incidence of syphilis in Nantong City showed an increasing trend, mainly manifested as an increase in latent syphilis, and the reported incidence of syphilis in various towns (streets) showed obvious spatial clustering, and attention should be paid to key areas and targeted interventions should be formulated.

## Introduction

Syphilis is a chronic sexually transmitted infection (STI) caused by *Treponema pallidum*. Globally, syphilis is still a widespread and burdensome disease [1, 2]. In the past few years, the incidence of syphilis has been on the rise worldwide, although the route of transmission of syphilis is well known [3]. Delayed treatment after syphilis infection can lead to multiple organ damage. In addition to the physical symptoms caused by itself, it also increases the risk of acquiring HIV [4].

Syphilis, gonorrhea, genital *chlamydia*, trachomatis infection, genital herpes and condyloma acuminatum are the five STIs currently monitored in China. The monitoring data showed that the number of reported cases of syphilis increased year by year from 2013 to 2019, followed by fluctuating changes, but the overall trend was still on the rise [5]. Since 2007, the reported incidence of syphilis in Jiangsu Province, China, has been second only to tuberculosis and hepatitis B, with more than 20,000 cases reported annually from 2010–2016 [6, 7]. Nantong City is located in the southeast of Jiangsu Province in China, northeast of the Yangtze River Delta, south of the Yangtze River, east of the Yellow Sea. With the rapid development and the large scale of population flow, the pressure of prevention and control is increasing [8]. Surveillance data in Nantong City showed that the number of reported cases of syphilis has been ranked first among the five monitored STIs [9].

The spatial epidemiology method can visually display the distribution characteristics of diseases by mapping, and is now more and more widely used in disease analysis [10]. However, few studies have used spatial epidemiological methods to analyze the prevalence of syphilis in Jiangsu Province, especially at a more refined town (street) scale. Therefore, the purpose of this study is to analyze spatio-temporal distribution characteristics and changing trends of syphilis epidemic in Nantong City, and to provide reference for formulating more accurate prevention and control strategies.

## Methods

### Data Collection and Management

Data on syphilis cases were obtained from the Disease Surveillance Module of the China Information System for Disease Control and Prevention, and all cases were diagnosed and confirmed in accordance with the Diagnostic Criteria for Syphilis issued by the Ministry of Health of the People’s Republic of China. Diagnosis of five different types of syphilis refer to WS 273-2007 “Diagnostic Criteria for Syphilis” [11] and after 1 August 2018 refer to WS 273-2018 “Diagnostic Criteria for Syphilis” [12].

Population data were obtained from Nantong Bureau of Statistics. Vector map data were obtained from the National Geographic Information Resources Catalog Service System.

Excel 2020 was applied to establish the syphilis case database, and all case information was anonymized to protect patient privacy. In our study, the geographic data corresponds to the reported residence address of the individuals at the time of diagnosis.

### Materials

Joinpoint 5.0.1 software was used to analyze the trend of reported incidence of syphilis in Nantong from 2013 to 2022. ArcGIS 10.2 software was used to analyze the spatial autocorrelation of the reported incidence of syphilis in each town (street) of Nantong City. SaTScan10.1 was used for spatio-temporal scanning to explore the aggregation from time and space dimensions. In this study, *p* < 0.05 was used to determine statistical significance. Before 2018, towns (streets) were re-planned and adjusted. Therefore, the data used in the analysis of spatial and temporal laws are 2018–2022.

### Joinpoint Regression Model

The basic principle of Joinpoint regression analysis is to generate several nodes for a long-term trend through model fitting, and finally form a statistically significant trend segment [13, 14]. Grid Search Method is usually used to find nodes and determine the number and location of nodes. Annual Percent Change and Average Annual Percent Change (AAPC) were used to describe the trend. Trends are described using Annual Percent Change (APC) and Average Annual Percent Change (AAPC) [15].

### Global Spatial Autocorrelation

Using the “Global Moran’s I″ module of ArcGIS 10.2 software, we selected Global Moran’s I to explore whether there was spatial aggregation of syphilis incidence at towns (streets) level in Nantong City. The value of Global Moran’s I is between [−1, 1], greater than 0 means that the data are spatially positively correlated, less than 0 means that they are negatively correlated, and when it is equal to 0, the spatial distribution is randomized [16, 17].

### Local Spatial Autocorrelation

Using the “Anselin Local Moran’s I″ module of ArcGIS 10.2 software, we can further detect the areas and types of aggregation. The local Moran’s I is similar to the global Moran’s I, and when the value is greater than 0 and passes the statistical test, it indicates that the attribute value of the spatial unit is similar to that of the neighboring units (high-high cluster or low-low cluster), and when the local Moran’s I is less than 0, it indicates that the attribute value of the spatial unit is different from that of the neighboring units (high-low cluster or low-high cluster) [18]. “High values” refer to the attribute values of a region that are above the overall mean, while “low values” refer to attribute values that are below the overall mean [19].

### Spatio-Temporal Scanning

The Poisson model in SaTScan10.1 software was used to detect the aggregation of reported incidence of syphilis in towns (streets) of Nantong from two dimensions of time and space. SaTScan scans the target area dynamically with a circular window. The maximum scanning radius is 50% of the number of cases. Log-likelihood ratio (*LLR*) and relative risk (*RR*) were used to evaluate the risk intensity.

## Results

### Trend Analysis of Reported Incidence of Syphilis

The results of Joinpoint regression analysis showed that the total reported incidence of syphilis in Nantong from 2013 to 2022 showed an increasing trend (AAPC = 6.60%, *t* = 6.63, *p <* 0.01). Among them, the reported incidence of latent syphilis showed an upward trend (AAPC = 13.45%, *t* = 6.11, *p* = 0.01). The reported incidence of congenital syphilis showed a downward trend (AAPC = −24.01%), but there was a node in 2016. The APC of the reported incidence of congenital syphilis was-28.66% (*t* = 4.91, *p* < 0.01) in 2013–2016 and-6.54% (*t* = 3.65, *p* = 0.01) in 2017–2022. The reported incidence of secondary syphilis also showed a downward trend (AAPC = −5.03%, *t* = −5.76, *p <* 0.01). The annual change trend of Primary syphilis and Tertiary syphilis was not obvious, and the difference was not statistically significant (Table 1).

**TABLE 1:** Reported syphilis cases (incidence rate) and trend analysis in Nantong City, 2013–2022 (1/100,000) (Nantong City, Jiangsu Province, China, 2013-2022).

Year	Latent syphilis	Primary syphilis	Secondary syphilis	Tertiary syphilis	Congenital syphilis	Total
2013	904 (11.92)	350 (4.61)	524 (6.91)	16 (0.21)	28 (0.37)	1822 (24.02)
2014	1,263 (16.55)	485 (6.35)	586 (7.68)	29 (0.38)	29 (0.38)	2,392 (31.34)
2015	1,573 (20.50)	377 (4.91)	509 (6.63)	34 (0.44)	20 (0.26)	2,513 (32.76)
2016	1966 (25.59)	286 (3.72)	416 (5.41)	26 (0.34)	12 (0.16)	2,706 (35.22)
2017	2,152 (27.99)	285 (3.71)	438 (5.70)	31 (0.40)	10 (0.13)	2,916 (37.92)
2018	2,398 (31.16)	306 (3.98)	444 (5.77)	29 (0.38)	4 (0.05)	3,181 (41.34)
2019	2,346 (30.37)	286 (3.70)	420 (5.44)	19 (0.25)	3 (0.04)	3,074 (39.80)
2020	2,411 (31.20)	361 (4.67)	412 (5.33)	26 (0.34)	4 (0.05)	3,214 (41.59)
2021	3,113 (40.26)	401 (5.19)	397 (5.13)	34 (0.44)	8 (0.10)	3,953 (51.12)
2022	2,861 (36.95)	210 (2.71)	320 (4.13)	25 (0.32)	2 (0.03)	3,418 (44.14)
AAPC (95%*CI)*	13.45 (8.95, 18.14)	−3.80 (−8.95, 1.64)	−5.03 (−6.97, −3.05)	1.56 (−4.65, 8.18)	−24.01 (−32.31, −14.71)	6.60 (4.26, 8.90)
*t*	6.11	−1.62	−5.76	0.57	−5.48	6.63
*p*	0.01	0.14	<0.01	0.59	<0.01	<0.01

A further trend analysis of the reported incidence of syphilis in each district (county) of Nantong City from 2013 to 2022 showed that the reported incidence of syphilis in Rudong County, Rugao City, Hai ’an City and Tongzhou District showed an increasing trend, with an average annual rate of 13.31%, 12.32%, 10.59% and 7.31%, respectively. The reported incidence of syphilis in Qidong City, Haimen District, and Chongchuan District did not show any statistically significant trend. The overall trend in Nantong Development Zone was also not statistically significant, but there was a node in 2018, which showed an upward trend from 2013 to 2018, rising at a rate of 14.70% (*t* = 2.81, *p* = 0.01), and there was no statistically significant from 2018 to 2022 (Table 2).

**TABLE 2:** Reported syphilis cases (incidence rate) and trend analysis by district in Nantong City, 2013–2022 (1/100,000) (Nantong City, Jiangsu Province, China, 2013-2022).

Year	Chongchuan District	Nantong development zone	Haimen District	Tongzhou District	Rugao District	Qidong District	Haian District	Rudong Country
2013	423 (43.16)	62 (30.36)	204 (22.61)	259 (22.68)	183 (14.54)	339 (35.35)	157 (18.13)	195 (19.8)
2014	522 (53.03)	81 (39.28)	304 (33.69)	321 (28.11)	289 (22.99)	379 (39.61)	169 (19.51)	327 (33.27)
2015	513 (51.90)	76 (36.63)	307 (33.99)	385 (33.71)	233 (18.57)	497 (52.04)	233 (26.9)	269 (27.39)
2016	466 (46.93)	72 (34.45)	282 (31.17)	453 (39.67)	525 (41.95)	394 (41.34)	279 (32.21)	235 (23.93)
2017	552 (55.34)	124 (58.68)	296 (32.69)	495 (43.35)	561 (44.93)	407 (42.76)	217 (25.06)	264 (26.91)
2018	621 (61.85)	131 (60.54)	346 (38.22)	531 (46.50)	531 (42.66)	471 (49.53)	192 (22.20)	358 (36.55)
2019	517 (51.06)	137 (60.73)	339 (37.44)	500 (43.83)	548 (44.25)	412 (43.38)	252 (29.17)	369 (37.74)
2020	509 (46.27)	139 (49.34)	305 (32.14)	520 (43.35)	503 (40.73)	385 (40.17)	356 (40.98)	497 (53.52)
2021	796 (66.51)	152 (45.70)	311 (31.34)	647 (51.37)	520 (42.18)	440 (45.62)	481 (55.14)	606 (68.87)
2022	632 (52.24)	152 (45.67)	242 (24.37)	510 (40.48)	537 (43.78)	417 (43.37)	369 (42.41)	559 (63.56)
AAPC (95%*CI)*	1.89(−1.28, 5.17)	4.50 (−3.56, 13.22)	−0.76 (−7.60, 6.58)	7.31 (2.99, 11.81)	12.32 (1.76, 23.99)	1.08 (1.80, 4.05)	10.59 (5.05, 16.41)	13.31 (7.82, 19.07)
*t*	1.37	1.08	−0.21	3.06	2.31	0.86	4.52	5.80
*p*	0.21	0.28	0.83	<0.01	<0.01	0.42	<0.01	<0.01

### Global Spatial Autocorrelation

Global spatial autocorrelation analysis of the reported incidence of syphilis among towns (streets) in Nantong City from 2018 to 2022 showed that Moran’s I was positive and the *p*-values were all less than 0.05, indicating that the reported incidence among towns (streets) did not obey a random distribution (Supplementary Material S1).

### Local Spatial Autocorrelation

**FIGURE 1:**
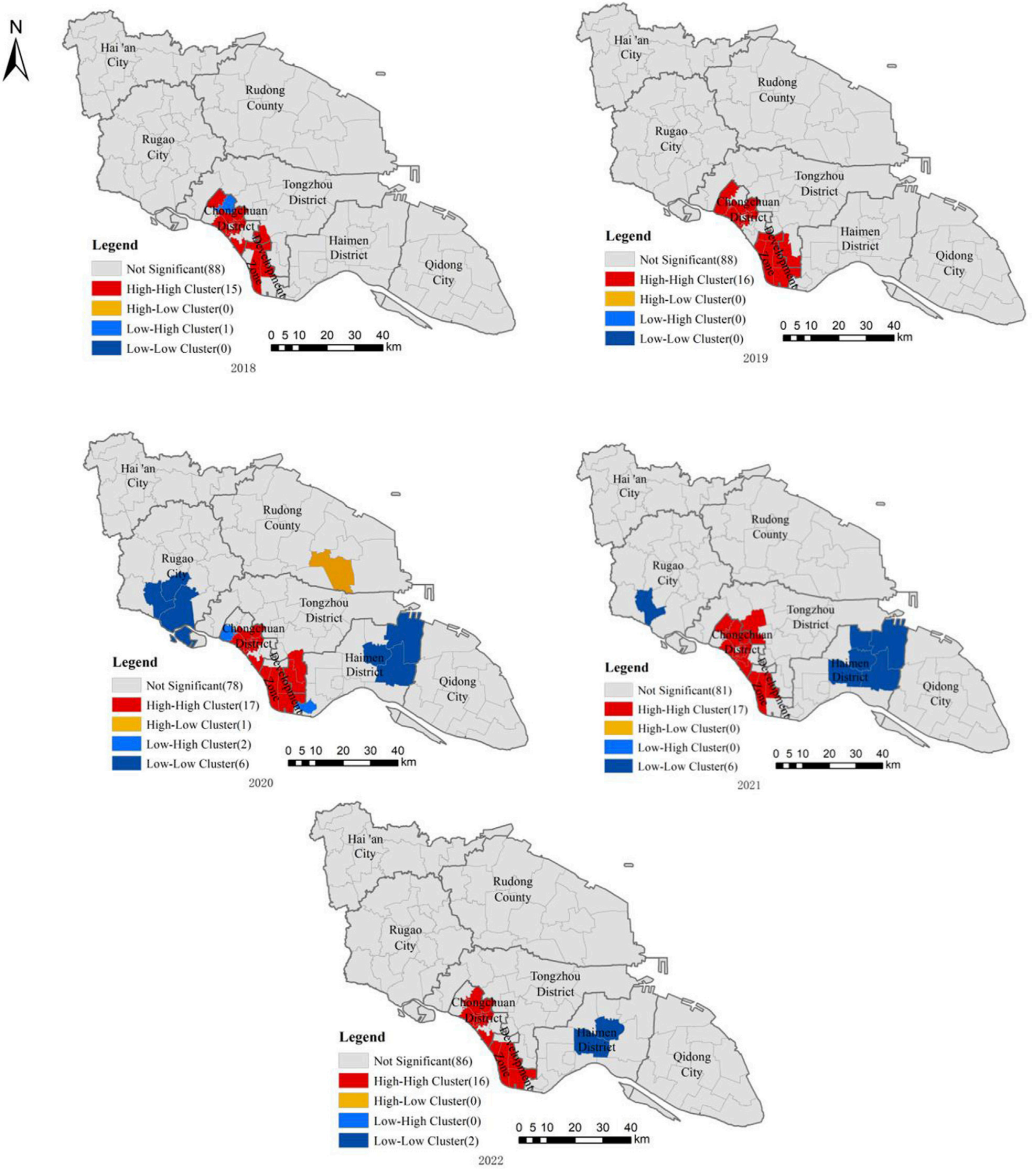
Local spatial autocorrelation analysis of reported incidence of syphilis in Nantong City, 2018–2022 (Nantong City, Jiangsu Province, China, 2018-2022).

The results of local autocorrelation analysis showed that the high-high clustering areas of syphilis reported incidence in Nantong City from 2018 to 2022 were mainly distributed in Chongchuan District and Nantong Development Zone, and spread to some streets in Tongzhou District. The high-low aggregation area only appeared in 2020 and was distributed in Juegang Street, Rudong City. The low-high aggregation area appeared in 2018 and 2020. In 2018, it was located in Xingfu Street, Chongchuan District. In 2020, it was distributed in Tianshenggang Street, Chongchuan District and Jiangxinsha Farm, Haimen District. The low-low aggregation area appeared in 2020–2022, mainly distributed in Haimen District and Rugao City (Figure 1; Supplementary Material S2).

### Spatio-Temporal Scanning

**FIGURE 2:**
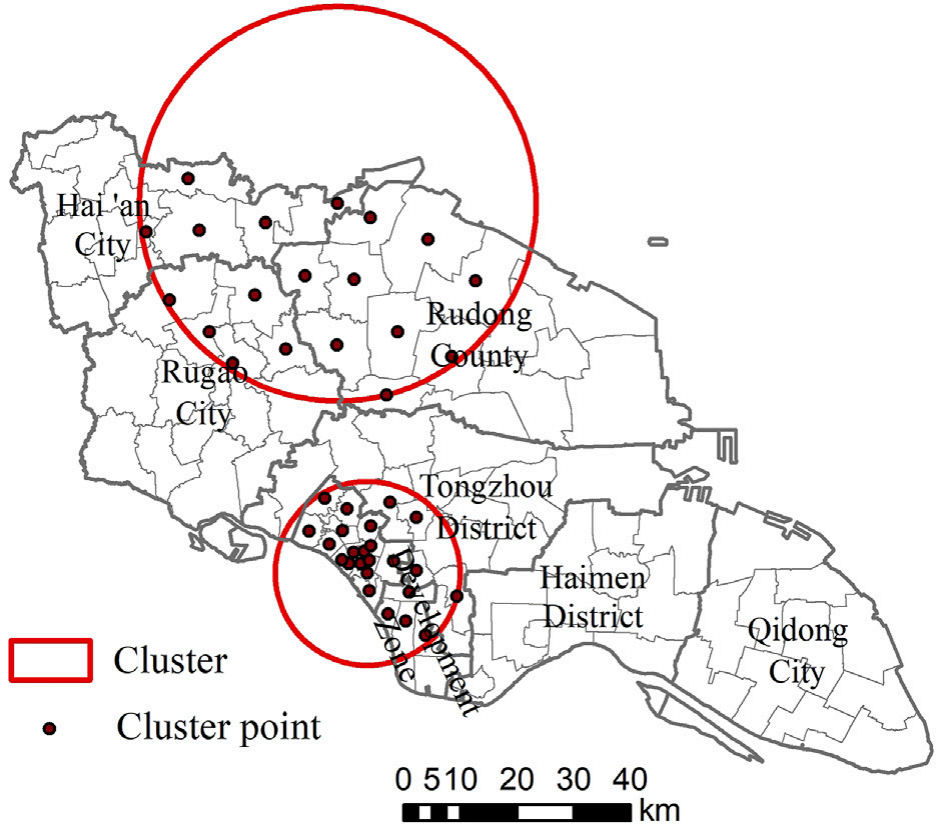
Spatio-temporal scanning cluster area of reported incidence of syphilis in Nantong City, 2018–2022 (Nantong City, Jiangsu Province, China, 2018-2022).

The results of spatio-temporal scanning showed that two aggregation areas were detected in 2018–2022, and the aggregation years were both 2021–2022. The first aggregation area contained 24 streets with a radius of 16.27 km, and the aggregation center was located in Wenfeng Street in Chongchuan District. The second gathering area includes 18 streets with a gathering radius of 34.90 km, and the gathering center is Jiaoxie Town, Hai’an City (Supplementary Material S3). The results with statistically significant differences were visualized in ArcGIS 10.2 software (Figure 2).

## Discussion

In this study, we used a combination of Joinpoint regression modeling and spatial epidemiology (spatial autocorrelation analysis and SaTScan spatio-temporal scanning) to study the reported incidence pattern of syphilis in Nantong City. Firstly, Joinpoint regression analysis was used to obtain the trend of reported incidence of syphilis in Nantong from 2013 to 2022. Then, spatial autocorrelation and spatio-temporal scanning analysis were used to detect the spatio-temporal variation of syphilis incidence in 5 years (2018–2022). Spatial autocorrelation analysis can detect whether there is spatial dependence in the observation data of the target area, and then show this dependence feature [20]. SaTScan spatio-temporal scanning does not make assumptions in advance for the aggregation area, and can maximize the use of data features [21]. It can detect aggregation in time and space, and visually display the aggregation area.

From the results of Joinpoint regression analysis, it can be seen that the reported incidence of syphilis in Nantong City from 2013 to 2022 showed an upward trend, which was mainly reflected in the increase in the reported incidence of latent syphilis. The trend of secondary syphilis showed a downward trend, and the trend of primary syphilis and tertiary syphilis was not obvious. The incidence trend of syphilis in each district (county) was analyzed. The results showed that the change trend of syphilis in Chongchuan District was not obvious, but the reported incidence had been at a high level. From 2013 to 2018, the reported incidence rate in Nantong Development Zone has been on the rise, and has remained stable since then. The incidence rate in Tongzhou District, Rugao City, Hai ’an City and Rudong County showed an obvious upward trend, while the trend in Haimen District and Qidong City was not obvious. Patients with latent syphilis do not show specific symptoms, but persistently carry the pathogen and are an important source of infection [22]. Primary syphilis typically presents with chancre symptoms 2–4 weeks after infection. If treated promptly and effectively, such as with penicillin, it can prevent the disease from progressing to secondary syphilis. Inadequate or absent treatment may lead to the advancement of the disease to tertiary syphilis [1]. The increase in latent syphilis and the decrease in secondary syphilis reflect the dynamics of syphilis diagnosis and prevention efforts [23]. The increase in the reported incidence of latent syphilis is closely related to the expanded screening of syphilis [24]. In 2010, the Ministry of Health of China promulgated the “China Syphilis Prevention and Control Plan (2010–2020),” and Jiangsu Province added a comprehensive STI control plan on this basis [6]. According to the plan, Nantong City has actively increased the training of STI diagnosis and treatment personnel in medical institutions within its jurisdiction. Active detection of syphilis was carried out, and STI patients and those with a history of high-risk sexual behavior were actively recommended for testing. The free counseling and testing of syphilis is included in the daily service content of AIDS voluntary counseling and testing and community drug maintenance treatment clinics, so the proportion of latent syphilis is increased. On the other hand, it has strengthened the publicity and education of key groups and the general population, promoted the correct medical behavior, promoted early diagnosis and treatment, and avoided the development of late syphilis [6].

The results of spatial autocorrelation analysis showed that there was a significant spatial positive correlation between the reported incidence of each street in Nantong City from 2018 to 2022. The range of high-high aggregation areas has not changed much in 5 years, involving 15 streets in Chongchuan District, all streets in Nantong Development Zone and four streets in Tongzhou District. Chongchuan District is located in the main urban area of Nantong City. The secondary and above medical institutions are relatively concentrated, and the medical opportunities and overall medical level are higher than other districts and counties. At the same time, factors such as more developed economy, large population mobility and high residential density in urban villages all affect the reported incidence of syphilis in the jurisdiction higher than other districts (counties) [25]. The geographical location of Nantong Development Zone is close to Chongchuan District. There are many factories in the area, attracting a large number of migrant workers. The large scale of population flow also makes the incidence of syphilis reported high. The results of SaTScan spatio-temporal scanning showed that two aggregation areas were detected in Nantong City from 2018 to 2022. The first aggregation area was consistent with the high-high aggregation area of spatial autocorrelation analysis. The second aggregation area included nine streets in Rudong County, four streets in Hai ‘an City and five streets in Rugao City. The SaTScan spatio-temporal scanning detects a larger range of aggregation areas, which can detect some secondary aggregation areas outside the main aggregation areas, maximize the detection of possible aggregation areas, and provide more information [21]. The identification of risk factors affecting syphilis in key streets should be strengthened, and targeted intervention measures should be formulated according to the characteristics of high-risk areas.

### Limitations

There are some limitations in this study. Since latent syphilis has no clinical manifestations, the discovery and reporting of latent syphilis cases included in this study rely on serological testing, which cannot fully represent the new incidence of the year [26]. Early latent syphilis can represent new cases. At present, the report of latent syphilis infectious diseases in China does not distinguish between early and late stages. Considering the large proportion of latent syphilis, abandoning this part of the data may weaken the epidemic situation. Therefore, this study included all stages of syphilis cases.

### Conclusion

In summary, the reported incidence of syphilis in Nantong City is on the rise, mainly manifested in the increase of latent syphilis reports, and the reported incidence in towns (streets) is significantly spatially clustered. By exploring the incidence trend of diseases and the characteristics of high-risk areas, this study has an in-depth understanding of the transmission characteristics of infectious diseases, so as to lay a foundation for formulating more accurate prevention and intervention measures.

## Abbreviations

STI, sexually transmitted infection; APC, annual percent change, AAPC, average annual percent change, LLR, Log-likelihood ratio, RR, relative risk.
