# Supplementary material for: Spatio-Temporal Distribution Characteristics of Syphilis: on the Scale of Towns (Streets) in Nantong City, Jiangsu Province, China
Source: Int J Public Health. 2025 Mar 18;70:1606875. doi: 10.3389/ijph.2025.1606875 (PMC11957987; doi:10.3389/ijph.2025.1606875)
Supplement: Supplementary file 1 [file Table1.DOCX]

Additional file 1

Global Moran’s *I* of incidence reported by townships (streets) in Nantong City，2018 -2022

| Year | Moran’s *I* | *z* | *p* |
| --- | --- | --- | --- |
| 2018 | 0.40 | 7.07 | 0.00 |
| 2019 | 0.42 | 7.30 | 0.00 |
| 2020 | 0.37 | 6.36 | 0.00 |
| 2021 | 0.57 | 9.66 | 0.00 |
| 2022 | 0.29 | 5.81 | 0.00 |
