# Supplementary material for: Spatio-Temporal Distribution Characteristics of Syphilis: on the Scale of Towns (Streets) in Nantong City, Jiangsu Province, China
Source: Int J Public Health. 2025 Mar 18;70:1606875. doi: 10.3389/ijph.2025.1606875 (PMC11957987; doi:10.3389/ijph.2025.1606875)
Supplement: Supplementary file 2 [file Table2.DOCX]

Additional file 2

Local spatial autocorrelation analysis of reported incidence of syphilis in towns (streets) in Nantong City ,2018 -2022

| Year | High-High | High-Low | Low-High | Low-Low |
| --- | --- | --- | --- | --- |
| 2018 | Chongchuan District: Chenqiao Street, Yongxing Street, Tangzha Street, Qinzao Street, Rengang Street, Hongqiao Street, Xinchengqiao Street, Xuetian Street, Chengdong Street, Zhongxiu Street, Langshan Street.  Nantong Development Zone: Xiaohai Street, Xinkai Street, Zhuhang Street.  Tongzhou District: Xianfeng Street. | - | Chongchuan District: Xingfu Street | - |
| 2019 | Chongchuan District: Tianshenggang Street, Chenqiao Street, Yongxing Street, Tangzha Street, Rengang Street, Hongqiao Street, Xinchengqiao Street, Chengdong Street, Zhongxiu Street, Qinzao Street.  Nantong Development Zone: Xiaohai Street, Zhongxing Street, Xinkai Street, Zhuhang Street, Jianghai Street.  tongzhou District: Zhangzhishan Town. | - | - | - |
| 2020 | Chongchuan District: Yongxing Street, Tangzha Street, Qinzao Street, Rengang Street, Hongqiao Street, Hepingqiao Street, Chengdong Street, Zhongxiu Street, Xinchengqiao Street, Xuetian Street, Langshan Street.  nantong Development Zone: Zhongxing Street, Xinkai Street, Zhuhang Street, Jianghai Street.  tongzhou District: Zhangzhishan Town, Chuanjiang Town. | Rudong County: Juegang Street | Chongchuan District: Tianshenggang Street  Haimen District: Jiangxinsha Farm | Haimen District: Yudong Town, Baochang Town, Yuelai Town.  Rugao City: Shizhuang Town, Wuyao Town, Changjiang Town |
| 2021 | Chongchuan District: Chenqiao Street, Yongxing Street, Tangzha Street, Qinzao Street, Xingfu Street, Rengang Street, Hongqiao Street, Xinchengqiao Street, Xuetian Street, Chengdong Street, Zhongxiu Street, Wenfeng Street, Langshan Street.  Nantong Development Zone: Zhongxing Street, Xinkai Street and Zhuhang Street.  Tongzhou District: Xingren Town | - | - | Haimen District: Yudong Town, Baochang Town, Yuelai Town, Changle Town, Zhengyu Town.  Rugao: Shizhuang Town. |
| 2022 | Chongchuan District: Yongxing Street, Tangzha Street, Xingfu Street, Qinzao Street, Rengang Street, Hepingqiao Street, Chengdong Street, Zhongxiu Street, Hongqiao Street, Xinchengqiao Street, Xuetian Street, Langshan Street.  Nantong Development Zone: Zhongxing Street, Xinkai Street, Zhuhang Street, Jianghai Street. | - | - | Haimen District: Changle Town, Yudong Town |
