# Supplementary material for: Spatio-Temporal Distribution Characteristics of Syphilis: on the Scale of Towns (Streets) in Nantong City, Jiangsu Province, China
Source: Int J Public Health. 2025 Mar 18;70:1606875. doi: 10.3389/ijph.2025.1606875 (PMC11957987; doi:10.3389/ijph.2025.1606875)
Supplement: Supplementary file 3 [file Table3.DOCX]

Additional file 3

Spatial-temporal scan results of reported incidence of syphilis in Nantong City, 2018 -2022

| Grade | Aggregation  time | Cluster center | | | Cluster | *LLR* | *RR* | *p* |
| --- | --- | --- | --- | --- | --- | --- | --- | --- |
|  |  | Latitude | Longitude | Radius |  |  |  |  |
| 1 | 2021-2022 | 31.990848 | 120.881988 | 16.27 | Chongchuan District: all streets.  Nantong Development Zone: Xiaohai Street, Zhongxing Street, Zhuhang Street, Xinkai Street.  Tongzhou District: Xianfeng Street, Xingren Town, Xingdong Street, Chuanjiang Town | 441.75 | 2.19 | <0.05 |
| 2 | 2021-2022 | 32.578314 | 120.81203 | 34.90 | Rudong County: Xindian Town, Matang Town, Fengli Town, Yangkou Town, Chahe Town, Shuangdian Town, Hekou Town, Bencha Town, Yuanzhuang Town.  Hai 'an City: Hai 'an Street, Chengdong Town, Libao Town, Jiaoxie Town.  Rugao City: Chengbei Street, Rucheng Street, Chengnan Street, Dingyan Town, Dongchen Town | 38.46 | 1.26 | <0.05 |
